# Supplementary material for: Multidimensional heritability analysis of neuroanatomical shape
Source: Nat Commun. 2016 Nov 15;7:13291. doi: 10.1038/ncomms13291 (PMC5116071; doi:10.1038/ncomms13291)
Supplement: Supplementary Information — Supplementary Figures 1-3 and Supplementary Table 1 [file ncomms13291-s1.pdf]

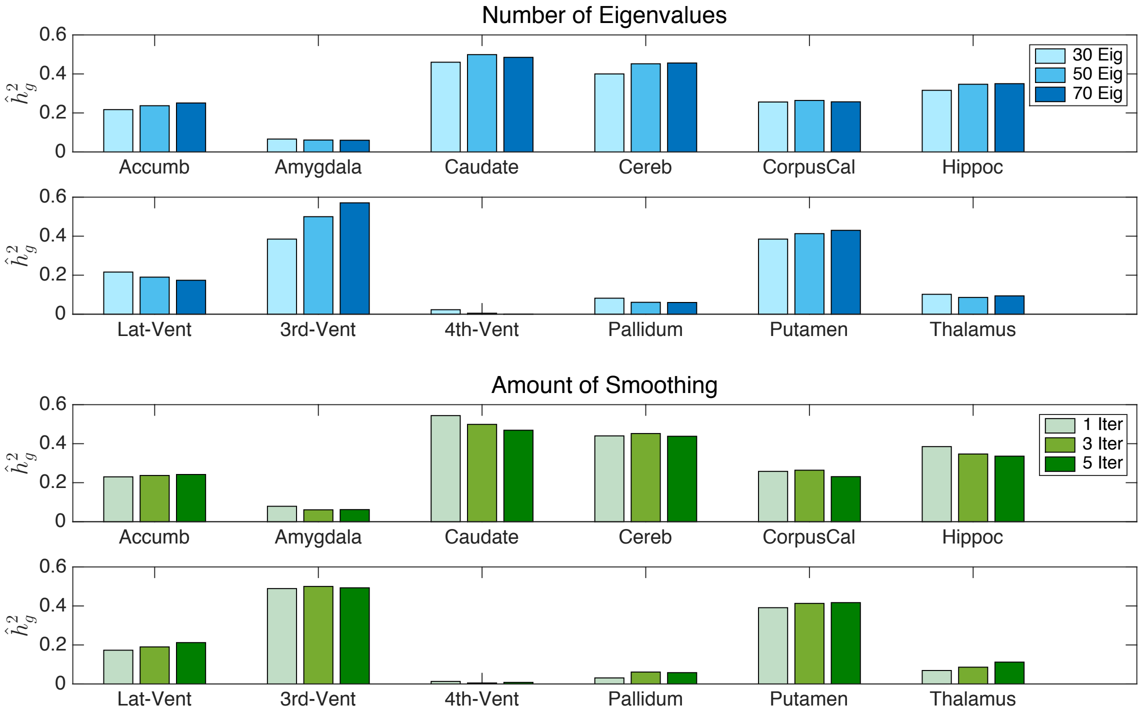

Supplementary Figure 1: SNP heritability estimates of the shape of brain structures using the GSP sample when incorporating different numbers of eigenvalues in the Laplace-Beltrami Spectrum (LBS) based shape descriptor with 3 iterations of geometric smoothing (upper panel), and when applying different amount of smoothing to the surface mesh representing the geometry of the object with the number of eigenvalues fixed at 50 (lower panel). The heritability estimates are largely robust to these parameter settings.

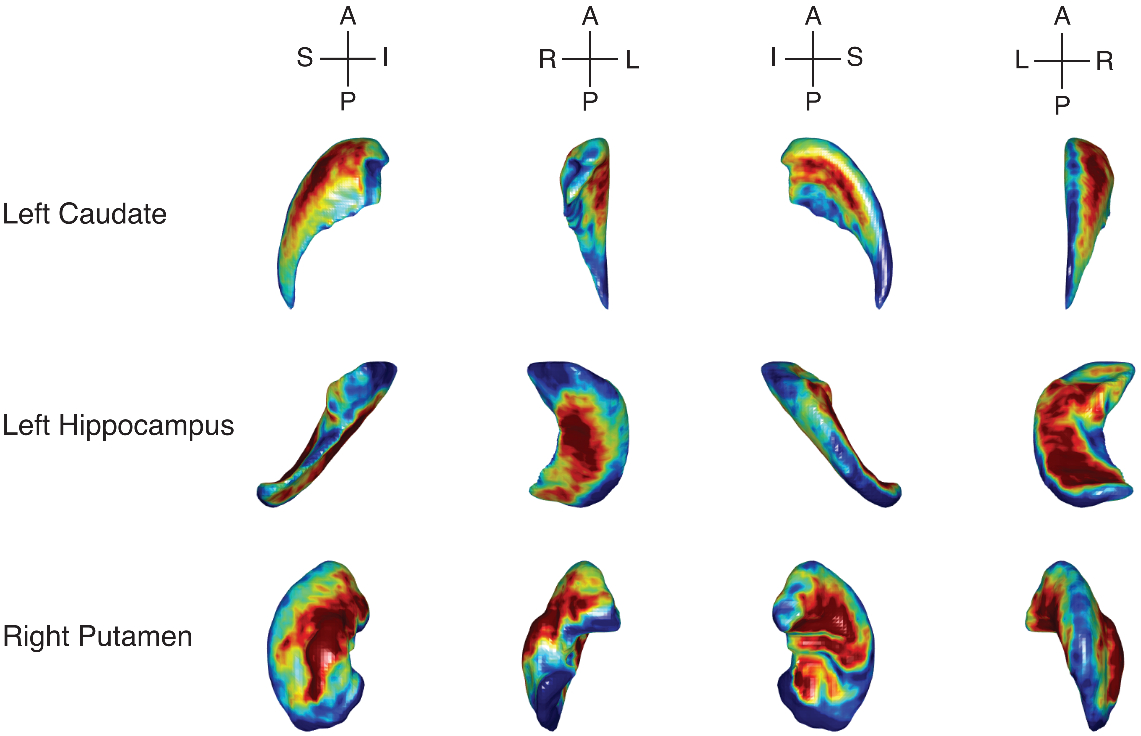

Supplementary Figure 2: The principal mode of shape variation for the contralateral brain structures of those reported in the main text. Each structure is represented with a sample-specific population average, on which average shapes at the two extremes (± 2 standard deviation or SD) along the first principal component (PC) of the shape descriptor (-2 SD, blue; $+2$ SD, red) are depicted. Anatomical orientation is indicated with embedded coordinate axes. I: Inferior, S: Superior, A: Anterior, P: Posterior, L: Left, R: Right. For the left caudate, the first PC explains 79% of the shape variation and has a SNP heritability estimate of 0.34. For the left hippocampus, the first PC explains 70% of the shape variation and has a SNP heritability estimate of 0.42. For the right putamen, the first PC explains 65% of the shape variation and has a SNP heritability estimate of 0.47.

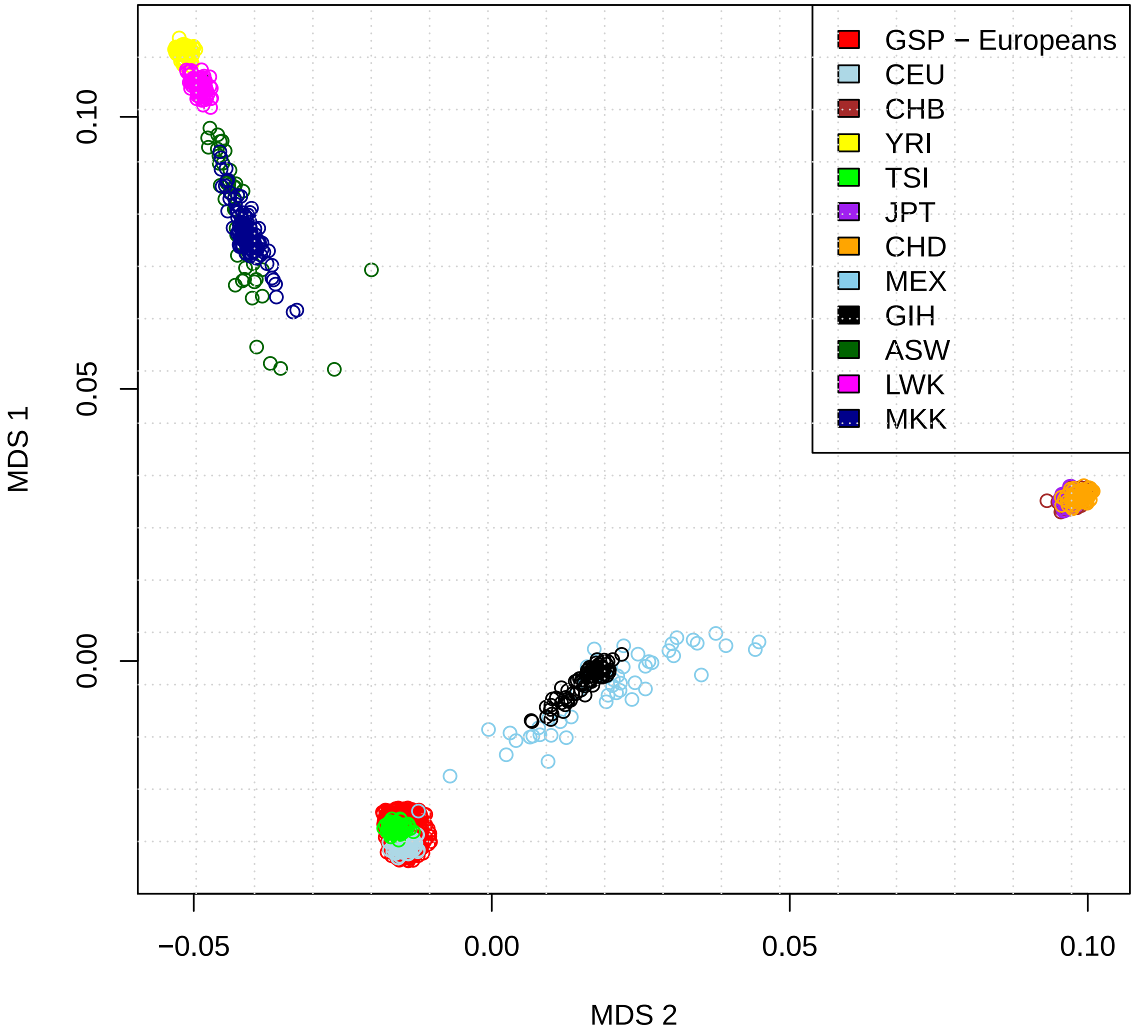

Supplementary Figure 3: Multidimensional scaling plot (MDS) for the GSP non-Hispanic European subjects, overlaid on samples from the 1000 Genomes Project.

Structure	Left			Right		
	$\widehat{h}^2_{\mathrm{SNP}}$	SE	Wald p-value	$\widehat{h}^2_{\mathrm{SNP}}$	SE	Wald p-value
Accumbens Area	0.256	0.182	0.080	0.255	0.180	0.078
Amygdala	0.140	0.189	0.229	0.001	0.174	0.500
Caudate	0.279	0.217	0.099	0.686	0.212	0.001
Hippocampus	0.308	0.200	0.062	0.391	0.199	0.025
Lateral Ventricle	0.160	0.206	0.218	0.230	0.192	0.115
Pallidum	0.218	0.151	0.075	0.001	0.159	0.500
Putamen	0.437	0.170	0.005	0.405	0.179	0.012
Thalamus Proper	0.001	0.182	0.500	0.195	0.174	0.132

Supplementary Table 1: SNP heritability estimates $\widehat{h}^2_{\mathrm{SNP}}$ of the shape of bilateral brain structures using the GSP sample, with their standard error (SE) estimates and Wald p-values.
